# Studying Healthcare Affordability during an Economic Recession: The Case of Greece

**DOI:** 10.3390/ijerph17217790

**Published:** 2020-10-24

**Authors:** Dimitris Zavras

**Affiliations:** Department of Public Health Policy, School of Public Health, University of West Attica, 11521 Athens, Greece; dzavras@uniwa.gr

**Keywords:** healthcare affordability, economic crisis, ability to make ends meet, capacity to cope with unexpected financial expenses, geographic characteristics, socioeconomic characteristics, epidemiologic characteristics, demand-side barriers, supply-side barriers

## Abstract

The significant deterioration of economic prosperity in Greece during the economic crisis decreased patients’ ability to pay. Thus, the objective of this study is to determine the factors affecting healthcare affordability in Greece during an economic recession. This study used data from the European Union Statistics on Income and Living Conditions (EU-SILC) 2016. The sample consisted of 18,255 households. Healthcare affordability was regressed on geographic characteristics as well as several variables that refer to the households’ financial condition. Region of residence, ability to make ends meet, and capacity to cope with unexpected financial expenses were found to be statistically significant. Using sample sizes of 1000 and 1096 adults, respectively, the European Quality of Life Surveys (EQLS) of 2007 and 2016 were also used as data sources. Economic crisis was expressed with a dummy variable: (1) 0: 2007, and (2) 1: 2016. Difficulty in responding to healthcare costs was regressed on survey year and several demographic, socioeconomic, and health characteristics, revealing that individuals were more likely to face difficulties in responding to healthcare costs during the economic crisis. These results confirm the mechanism on the basis of which economic crises affect healthcare access: primarily through the effects of demand-side barriers.

## 1. Introduction

Healthcare affordability, or, in other terms, “financial access”, is among the main dimensions of healthcare access and relates the healthcare services’ prices and providers’ insurance or deposit demands to users’ income, capacity to pay, and existing health insurance coverage [1]. Healthcare affordability may depend on a ratio of healthcare expenditure to non-healthcare expenditure with a consideration for budget constraints and other sourced elements of coverage [2]; the term “affordability” has no clear meaning in economics [3].

Thus, the operationalization of the concept of affordability requires: (i) informative data on household incomes; (ii) knowledge of the commodity price, and (iii) a definition of “unreasonable burden” [4]. However, there is a consensus that affordability is a subjective concept [5,6].

Healthcare affordability refers to financial and incidental costs [7] imposing barriers to access [8,9] and resulting in unmet healthcare needs [10], i.e., situations in which an individual needs healthcare but does not receive it [11]. Because both supply factors and demand factors result in unmet healthcare needs [12], unmet healthcare needs are a function of the healthcare system features or associated with the personal circumstances of those seeking healthcare [13]. In other words, unmet healthcare needs may be a consequence of limited availability of health services but may also arise, among others, from individual accessibility issues, such as cost [14].

The variables presented below are the key demand-side determinants of healthcare affordability, as they have been identified in the international bibliography.

By definition, healthcare affordability looks at prices of services as they relate to patients’ income and capacity to pay [15]. As such, affordability is associated with dimensions of poverty [16], as poverty directly negates access to material resources [17].

Since income and, to a lesser extent, occupation determine the resources of individuals [18], limited income, unemployment and lack of health insurance coverage are strongly related to affordability barriers [19]. That is, higher income as an indicator of the disposable financial means of individuals [20] directly increases healthcare affordability [21], while those with lower incomes often have restricted access to healthcare services [22]. Additionally, unemployment puts individuals at risk of poverty and because of its influence on material circumstances, it can impair financial healthcare access [23]. In addition to drastic income reduction, job loss is often linked to a loss of health insurance [24], especially in Bismarck-type social security schemes, although this is not the case in all healthcare systems. On the other hand, the existence of health insurance coverage can nearly eliminate the negative influence of limited income on healthcare access and by decreasing out-of-pocket payments, makes healthcare more affordable for lower income individuals [25], providing access that would be otherwise unaffordable [26].

Research shows that healthcare expenditures often compete with other expenses in family budgets. According to Kushel et al. (2006) [27], housing instability, i.e., difficulties in paying rent, mortgage, or utility bills, is considered a risk factor for reduced access to healthcare services. Thus, because high housing costs reduce disposable income and impede families’ capacity to account for other necessities [28], they constitute a risk factor for the postponement of healthcare services [29,30], which in essence translates to less overall investment in healthcare [31].

The relationship between health status, poverty and access to care is complex and confounded by several biologic as well as social determinants of health. Individuals with poor health often face unaffordable healthcare costs [32] due to their greater need for healthcare (more intense and more frequent use of healthcare), but also due to their low socioeconomic status. Poor health can result in fewer labor market opportunities and lower income as a result of lower productivity [33]. It is also well documented in social epidemiology that lower socioeconomic status and less disposable income force individuals to make life choices that lead to lower health status, creating a vicious cycle of increasingly poor health that leads to poor income and vice versa.

Age is another important variable in this discussion. In general, younger individuals are healthier than older adults but are more likely to be uninsured [34], and thus they face financial barriers in accessing healthcare [35] when they need it. However, the effect of age on healthcare affordability requires special analysis, since the probability of an individual being in poverty decreases from childhood to adulthood and then increases again with advanced age over one’s own life cycle [36].

Research on gender differences shows that females have more difficulty affording healthcare than males [37], because females are more vulnerable to gaps in medical insurance coverage [38], but also because they have less access to material resources as compared to males [39]. In addition, females are more likely to develop numerous chronic health conditions resulting in high costs [38], but also are more vulnerable to reduced incomes and unemployment, as mentioned above.

Place of residence is one more variable that is important in the analysis between income, healthcare affordability and access. Healthcare affordability is found to be reduced for residents of rural areas [40], perhaps because rural populations are poorer, earn less at work, and work in industries with lower levels of employer-sponsored healthcare insurance coverage [41].

**Figure 1:**
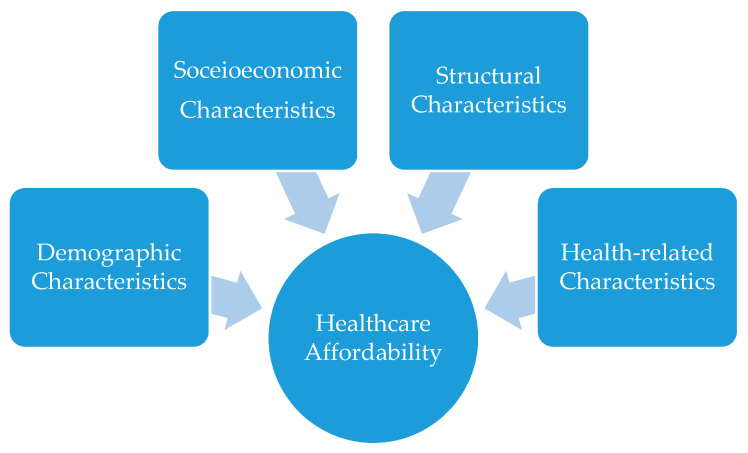
Main demand-side factors affecting healthcare affordability.

In summary, healthcare affordability depends on health systems features, such as financing [42], but it also depends on the population characteristics. Specifically, demographic (age and gender) and socioeconomic characteristics (income and occupation), health-related characteristics (health status, existence of a chronic health disease) and structural characteristics (health insurance coverage) affect healthcare affordability (Figure 1). Although demand-side factors such as those mentioned above are the main determinants of healthcare affordability at the individual level, the interrelation of these factors with the healthcare system should not be neglected. However, the causal relationship between healthcare systems with health and wealth-two of the most important determinants of healthcare affordability-is not clear cut and not easily measurable [43].

As mentioned above, healthcare access depends not only on individual or population characteristics, but also on healthcare system characteristics [44]. Access to care issues as a function of the characteristics of particular healthcare systems must also be studied. For example, exemptions and low-income protection in several healthcare systems guarantee access to certain vulnerable groups of the population, such as those in poor health, older individuals, children or adolescents, women, and low-income individuals [45]. That is, the factors that affect access are related to healthcare system design.

Although in theory most of the European countries provide universal or nearly universal population coverage, research shows that low-income individuals, those in poor health, those between 20 and 30 years old, the unemployed, and women have a higher probability of feeling unable to access care. That is, the most financial disadvantaged groups of the population are the most likely to feel that they will be unable to access needed care [46].

Indeed, it is evident that people in the lowest income quintile are almost three times more likely to forgo healthcare for financial reasons versus richer individuals. In most member countries of the Organization for Economic Co-operation and Development (OECD), healthcare is less affordable for households with a low income than those with a high income [47]. The same figure also stands for developing countries: the poor face more financial difficulties to access healthcare [48].

Among the countries facing the economic crisis of 2008, Greece experienced the most severe economic recession. Several fiscal measures and structural reforms were implemented after May 2010 and triggered high unemployment and a considerable reduction in disposable income.

Greece’s healthcare system is a mixed system in terms of both funding and provision-that is, a national health service type of system that coexists with a compulsory work-related social insurance system and a private healthcare sector. The National Organization for the Provision of Health Services (EOPYY) was established in 2011 after merging four large health insurance funds and was both a provider and purchaser of care. It covers the insured and their dependents. Since 2014, EOPYY has acted only as purchaser of care for the vast majority of the insured.

Insured individuals can access all public primary and secondary healthcare services free of charge; they also have access, on a case-by-case cost-sharing basis, to certain private providers contracted with EOPYY. In the case of private healthcare providers not contracted with EOPYY, insured individuals have to pay the entire cost by themselves (out-of-pocket payments) or through private insurance. Public healthcare coverage by EOPYY has been ensured since 2016 for uninsured citizens [49].

The significant deterioration of economic prosperity in Greece during the economic crisis decreased patients’ ability to pay [50]. Thus, a substantial percentage of the Greek population could not afford healthcare [51]. The abovementioned evidence reflects the fact that private spending is one of the main sources of healthcare funding in Greece; the other two are taxation and social insurance. That is, not only could few Greeks afford private healthcare [52], but a significant percentage of insured individuals also limited their use of the EOPYY units because they could not afford co-payments [53].

Based on the previous points, the objective of this study is to determine the factors affecting the affordability of healthcare services in Greece during an economic recession. A second goal is to determine the characteristics of those considering healthcare affordability as a reason for unmet healthcare needs. A third goal is to study the impact of the economic crisis on individuals’ ability to respond to their healthcare costs.

## 2. Materials and Methods

### 2.1. Data Sources

In this study, healthcare affordability was operationalized as households’ perceived ability to respond to the healthcare costs.

In order to study healthcare affordability, three different models were fitted. The first model, which attempts to determine the factors affecting the affordability of healthcare services in Greece during an economic recession, used data at the household level from the EU-SILC 2016. The second model, which attempts to determine the characteristics of those considering healthcare affordability as a reason for unmet healthcare needs, used data at the individual level from the EU-SILC 2016. The third model, which attempts to study the impact of the economic crisis on individuals’ ability to respond to the healthcare costs, used data at the individual level from the EQLS 2007 and 2016.

As mentioned above, in the first model and second model, data from the EU-SILC 2016 were used (Source: Hellenic Statistical Authority, EU-SICL 2016). The survey used a two-stage stratified sampling. The sample selection strata were based on the 2011 Census of the Hellenic Statistical Authority and were defined by region based on the Nomenclature of Territorial Units for Statistics II (NUTS II) and urbanity status. The sample size was 18,255 households. A total of 37,850 interviews were completed. All household members aged 16 years and over were selected for an interview. A personal interviewing technique was used for data collection. The data collection took place between May 2016 and November 2016.

In addition, in order to study the effect of the economic crisis on individuals’ ability to respond to the healthcare costs (third model), we used data from the EQLS of 2007 and 2016 (Source: United Kingdom Data Archive (UKDA), EQLS 2003–2016) [54]. EQLS 2007 used a multi-stage, stratified, and clustered design with a “random walk” procedure for the selection of the households at the last stage. The sample size was 1000 adults. A face-to-face interviewing technique was used for the data collection. The data collection took place between September 2007 and November 2007. EQLS 2016 used a multi-stage, stratified, random sampling design. The sample size was 1096 adults. A face-to-face interviewing technique was used for the data collection. The data collection took place between September 2016 and February 2017. Economic crisis was expressed with a dummy variable: (1) 0: 2007, and 1: 2016. The analysis was not based on weighted data because the weighting methods of EQLS 2007 and EQLS 2016 were different (Eurofound, 2020).

### 2.2. Dependent and Independent Variables Used in the Three Models

Potential predictors in the analysis were considered that: (a) are dependent on the economic crisis; and (b) have been identified in the international bibliography as determinants of healthcare affordability, unmet healthcare needs, and individuals’ ability to respond to the healthcare costs. An additional criterion of independent variable selection was variable availability. For example, health insurance coverage was not available in the EU-SILC 2016, EQLS 2007, and EQLS 2016 surveys.

#### 2.2.1. Variables Used in the First Model

The dependent variable in the first model was households’ perceived ability to afford to pay for healthcare or households’ perceived ability to respond to the healthcare costs; the question under study was asked as follows: “is your household able to afford to pay for health care services provided for all household members?”, with answers: (1) with great difficulty; (2) with difficulty; (3) with some difficulty; (4) fairly easily; (5) easily, and (6) very easily. That is, perceived ability to afford to pay for healthcare corresponds to a single item concerning the household as a whole. The question was asked to the person responding to the household questionnaire.

In several studies, the ability to pay for healthcare is used as a synonym of healthcare affordability [55,56,57,58]. This specific ability interacts with direct, indirect, and opportunity costs in order to generate access. Thus, affordability reflects the economic capacity for people to spend resources and time to use services [59].

The outcome was dichotomized [60]: 0 to denote difficulty (great difficulty; difficulty; or some difficulty; and 1 ease (fairly easily; easily; or very easily) [61]. Health care spending is identified as unaffordable if costs exceed a relative threshold determined by a family’s income or, more appropriately, a fraction of a family’s available resources [62]. The existence of the affordability threshold justifies the use of a dichotomous variable. In addition, several studies have focused on the presence or absence of financial barriers [63,64,65]. Furthermore, the ability to pay may be approached by a dichotomous variable [66]. Thus, a logistic regression model was fitted to determine the factors associated with the degree of cost-related difficulty facing Greek households with regard to healthcare use. The potential predictors used in the first model are presented in Table 1.

Densely populated areas are defined as those areas with at least 50% of people living in contiguous grid cells of 1 km^2^ with a density of at least 1500 inhabitants per km^2^ and a minimum population of 50,000. Intermediate density areas are defined as areas with clusters of contiguous grid cells of 1k^2^ with a density of at least 300 inhabitants per k^2^ and a minimum population of 5000. Thinly-populated areas are defined as areas with more than 50% of the population living in rural grid cells outside urban clusters (Eurostat, 2020).

#### 2.2.2. Variables Used in the Second Model

As mentioned above, data at the individual level (data source: EU-SILC 2006) were also analyzed. Specifically, each household member was asked two questions in terms of their healthcare use. The first question was, “was there any time during the past 12 months when you really needed medical examination or treatment (excluding dental) for yourself?” The potential answers to the first question were (1) no and (2) yes, at least one occasion. The 2nd question was “did you have a medical examination or treatment each time you really needed?” The potential answers to the second question were (1) yes (I had a medical examination or treatment each time I needed) and (2) no (there was at least one occasion when I did not have a medical examination or treatment). The respondents were then asked the question: “what was the main reason for not having a medical examination or treatment? with answers: (1) could not afford it (too expensive or there was no insurance coverage; (2) waiting list; (3) could not take time because of work, care for children, or for others, etc.; (4) too far to travel or no means of transportation; (5) fear of medical doctors, hospitals, examination, or treatment; (6) wanted to wait and see if the problem got better on its own; (7) did not know any good medical doctor; and (8) other reasons. This was dichotomized as: (a) 1: could not afford it (too expensive or there was no insurance coverage) and (b) 0: all the remaining reasons. Thus, a logistic regression model was performed, using the derived variable as the outcome. The potential predictors used in the second model are presented in Table 2.

#### 2.2.3. Variables Used in the Third Model

In order to study the effect of the economic crisis on individuals’ ability to respond to the healthcare costs, we used data from the EQLS of 2007 and 2016. The respondents were asked the question, ”thinking about the last time you needed to see or be treated by a General Practitioner (GP), family doctor, or health center, to what extent did any of the following make it difficult or not for you to do so?” With regard to the option “cost of seeing the doctor”, the answers were: (1) very difficult; (2) a little difficult, and (3) not difficult at all. This variable (the outcome) was dichotomized as follows: (1) 0 was not difficult at all, and 1 was a little difficult and very difficult. The rationale of dichotomization is as mentioned above, but it also was driven by the need for comparability with previous results. Since the outcome was dichotomized, a logistic regression model was fitted. The potential predictors used in the third model are presented in Table 3. Income was not used as a potential predictor due to the high percentage of missing values (19.37%).

### 2.3. Independent Variables’ Coding

Helmert coding was applied to the ordinal variables (a) 1st model: population density, ability to make ends meet, being in arrears on utility bills during the last 12 months, financial burden induced by total housing cost; (b) 2nd model: age, self-rated health, education and (c) 3rd model: degree of urbanity(subjective), self-rated health, education, ability to make ends meet). Helmert contrast compares each category (except the last) with the differences from the balanced mean of subsequent levels. Indicator coding was applied to the nominal variables (a) 1st model: region; (b) 2nd model: occupation and (c) 3rd model: occupation). Indicator contrast compares the reference category of a nominal variable with the remaining categories. Binary variables (a) 1st model: capacity to cope with unexpected financial expenses; (b) 2nd model: gender, existence of a chronic health condition and (c) 3rd model: year, gender, existence of a chronic health condition) were treated as such.

### 2.4. Multicollinearity

Multicollinearity was tested through the variance inflation factor (VIF) and the tolerance. The VIF should not be greater than 10, and the tolerance should not be less than 0.1. Multicollinearity was not present (in each model, VIF < 10 for all predictors, and tolerance > 0.1 for all predictors).

### 2.5. Fitting Process

The fitting process for all three models is as follows: although, the sample sizes of both EU-SILC 2016 and EQLS 2007 and 2016 surveys were large, the variable selection was based on a univariate model for each explanatory variable. Any variable with *p* < 0.25 was considered to be a candidate for the multivariate model. Once the variables were identified, a model containing all of the selected variables was fitted [67]. The healthcare affordability model had *p* < 0.25 for all potential predictors in the univariate analysis. For the unmet need due to affordability issues model, the existence of a chronic health disease had *p* > 0.25 (*p* = 0.873) in the univariate analysis. The remaining variables had *p* < 0.25. In the individuals’ ability to respond to the healthcare costs model, age (*p* = 0.371), occupation (*p* = 0.526), and subjective urbanity status (*p* = 0.336) had *p* > 0.25 in the univariate analysis. The other variables had *p* < 0.25. The models only included the statistically significant covariates (significance level was set to a = 0.05).

The calibration of the models was tested with the calibration belt test [68]. In addition, the models were tested for specification error through the link test [69]. McFadden’s $R^{2}$ [70] was then calculated.

The STATA 14 statistical software package was used for the analysis. More specifically, the commands desmat [71], collin (Author: Ender P. B.), logistic, linktest, calibrationbelt [72], and fitstat [73] tests were used.

## 3. Results

### 3.1. First Model

Most (71.25%) Greek households used healthcare services during the last 12 months. In addition, 89.52% of the Greek households made a payment (either fully or partially) for healthcare services during the last 12 months. Furthermore, a substantial percentage of the Greek population (31.97%) reported facing great difficulty in responding to healthcare costs, while a similar percentage (34.70%) reported facing difficulty (Table 4).

According to the logistic regression model (first model), healthcare affordability depends on the region of residence, the ability to make ends meet, and the capacity to face unexpected financial expenses. Residents of Attica found it easier to respond to the cost of healthcare versus residents of other regions. In the same vein, financially comfortable households and households that could face unexpected financial expenses reported a lower likelihood of cost-related difficulties with regard to healthcare use (Table 5).

The link test indicates that the model does not suffer from specification error (Table 6).

In addition, McFadden’s $R^{2}=0.30$ indicates a perfect fit [70]. Furthermore, the calibration belt test (p=0.096) indicates good calibration.

### 3.2. Second Model

With respect to unmet healthcare needs, 14.40% of the respondents did not receive needed healthcare during the last 12 months at least once. Most respondents (83.15%) reported unmet healthcare needs due to affordability issues (Table 7).

According to the logistic regression model (second model), the unmet healthcare need due to affordability issues depends on self-rated health, education, and occupation. Specifically, individuals with lower education are more likely to report unmet healthcare needs due to affordability issues. The same also holds for the unemployed, pupils, students, those in compulsory military or community service, those in further training or in unpaid work experience, and those fulfilling domestic tasks and care responsibilities relative to those working full-time. However, those rating their health as very bad and those rating their health as good are less likely to report an unmet healthcare need due to affordability issues versus the subsequent levels. Those rating their health as fair are more likely to report an unmet healthcare need due to affordability issues (Table 8).

The link test indicates that the model does not suffer from a specification error (Table 9).

McFadden’s $R^{2}=0.066$ indicates a poor fit [70]. However, the calibration belt test (p=0.083) indicates good calibration. Both models have a good fit based on the diagnostic tests.

### 3.3. Third Model

According to the EQLS 2007 and 2016 data, in 2007, the percentage of those facing difficulty in responding to the healthcare costs was 46.22%. This percentage was 64.85% in 2016 (Table 10).

According to the logistic regression model (third model) (Table 11), individuals were more likely to face difficulties in responding to the healthcare costs during the crisis (year = 2016). In addition, women were more likely to face difficulties in responding to the healthcare costs. However, those making ends meet very easily, easily, fairly easily, and with some difficulty were less likely to face difficulties in responding to the healthcare costs than those making ends meet with higher levels of difficulty.

According to the link test (Table 12), the model does not suffer from specification error. McFadden’s $R^{2}=0.057$ indicates a poor fit [70]. However, the calibration belt test (p=0.192) indicates good calibration.

## 4. Discussion

Affordability is commonly referred to as the “degree of fit” between the full costs (the price of service at the point of delivery as well as other direct or indirect costs) and the users’ capacity to pay within the framework of their budget and other demands on that budget [74].

As mentioned in the introduction, healthcare affordability is a subjective concept [75]. We note that uncertainty constitutes a source of subjectivity with regard to healthcare affordability because prices in the healthcare market are uncertain [76,77], and the same holds for both the health and the effectiveness of medical treatment [78]; thus, the price and quantity of medical services are not initially known [79]. The uncertainty surrounding the decision-making process-the process with regard to healthcare use [80]-refers to limited knowledge and involves subjectivity [81]. Furthermore, self-perceived affordability constitutes a subjective assessment [82].

At this point, we note that affordability is considered a subjective concept not only at the level of individual judgment but also at the level of political judgment [83].

Although several demographic, socioeconomic, structural, and health-related factors influence healthcare affordability, interpreting the effect of geographic characteristics in a consistent health economics framework requires considering both the economic characteristics of the areas under study and the regional distribution of healthcare services.

Thus, the area of residence can have a negative effect on healthcare affordability and may be partially explained by the fact that, in Greece, the best situation with regard to the risk of poverty is observed in Attica (Source: Eurostat, 2020). On the other hand, Greek resources are largely concentrated in Athens [84]. Because supply-side issues play an important role in limiting access [85], it is obvious that people who live further from healthcare services are likely to face higher costs related to the use of medical care than those living closer [86]. It is also evident that a substantial percentage of the Greek population cannot even afford the cost of transportation to healthcare services [87]; thus, accessibility influences the choice of services available and the costs-both monetary and non-monetary-that users must pay to gain access [88]. These findings justify the results of a survey conducted in northern and western Greece indicating that the financial means of a substantial percentage of these areas’ residents were not sufficient for them to afford healthcare [89].

The improvements in affordability in Greece requires interventions at the national level in terms of resource allocation, because accessibility overlaps with availability and includes affordability [90]; poor geographic availability of healthcare services impacts affordability [91].

The effect of the remaining variables that were found to be statistically significant is somewhat obvious. The ability to make ends meet is one of the dimensions of living standards [92] and is directly related to healthcare affordability [93]. It is obvious that the inability to make ends meet constitutes the primary determinant of relinquished healthcare due to cost in many countries [94]. That is, greater household capacity to meet economic needs lowers forgone medical care due to cost [95]. Those struggling to make ends meet do not have extra money to pay for unexpected illnesses [96]. Thus, access to affordable healthcare is a great concern for those struggling to make ends meet [97].

According to the bibliography, which is limited, individuals that cannot cope with unexpected expenses face higher healthcare costs [98]. A probable reason is that this group is likely to be in poor health [99]. Because households’ poor economic condition implies a burden in terms of healthcare access, those with limited capacity to face unexpected financial expenses are more inclined to report unmet healthcare needs due to cost [100]. On the other hand, the unpredictability of illness exposes individuals to unexpected expenses [101,102].

According the analysis, the results obtained from EU-SILC 2016 justify the results obtained from EQLS 2007 and 2016.

The empirical findings demonstrate a dramatic worsening of the living and welfare standards of many Greeks over the years of recession and austerity [103]. The reduction in economic prosperity during the crisis decreased the patients’ capacity to pay and therefore negatively impacted their ability to afford healthcare services [104,105]. On the other hand, as the state reduced its overall financial responsibility in healthcare, a substantial part of the cost was shifted to households and individuals [53]. Thus, while the Greek population is universally covered, healthcare remains unaffordable [106]. This situation justifies the effect of both demand-side and supply-side barriers to healthcare access [107].

What was described in the results section is quite similar to subsequent years, with the exemption of 2017. In 2017, among households that reported unmet healthcare needs, 74.66% cited cost as the main reason for not using needed healthcare. This percentage was 81.65% and 81.40% in 2018 and 2019, respectively. Nevertheless, the percentage of unmet healthcare needs increased compared to that of 2016 (2017: 24.50%; 2018: 23.34%, and 2019: 21.28%) (Source: Hellenic Statistical Authority, EU-SILC 2017, EU-SILC 2018, EU-SILC 2019). These findings confirm the argument that cost is a critical factor driving healthcare-seeking behavior [108].

The results of this study are consistent with a significant strand of the literature, not only with regard to the factors affecting healthcare affordability but also with regard to the characteristics of those reporting unmet healthcare needs due to affordability issues. Indeed, females (OR = 2.20), individuals suffering from at least one chronic disease (OR = 1.41), individuals with poor health (OR = 2.90), individuals with low income (OR for the first quintile = 2.67 and OR for the second quintile = 2.02), people facing economic difficulties (OR for the frequency of economic problems = 1.99 and OR for the degree of economic difficulties = 1.86) and individuals without a paid job (OR = 1.02) are more likely to report unmet healthcare needs for financial reasons. In addition, individuals of higher education (OR = 0.77), and older individuals (OR = 0.97) are less likely to report unmet healthcare needs for financial reasons [109,110,111].

Based on the results of this study, healthcare costs are a significant barrier for healthcare access in Greece.

This finding is also justified by the data of Eurobarometer 91.2 [112]. According to this survey, 42% of Greek respondents identified more affordable treatments as a way to improve healthcare access for all Europeans. This percentage is much higher than the European Union average (33%).

However, the results of this survey are somewhat surprising because this percentage is too high in countries such as the Netherlands (69%), where affordability-especially for those in a more financially difficult position-is considered to be very good [113]. In addition, this percentage is 29% in United Kingdom, operating a Beveridge healthcare system, while France’s Bismarck healthcare model has 30%. However, with regard to affordability, the United Kingdom is ranked first, while France is ranked second [114]. A similar percentage was reported in Germany (29%), where healthcare is considered to be quite affordable [115].

A probable reason for these inconsistencies may be that the question was not country-specific. That is, according to Mooney (2009) [116], the community’s judgement with regard to access barriers is based on putting themselves in the position of those facing these barriers. However, Mooney does not refer to financial barriers, because they apply to all. We argue that that this is not the case in this specific question because the respondents should consider the whole European population.

In the case of Greece, the results are in line with the fact that 48% of the respondents declared unemployment as one of the two essential issues dealing with the country, while 45% declared the economic situation to be one of the two essential issues dealing with the country. Nevertheless, only 12% declared health and social security to be an issue.

However, the EU-SILC 2016 data confirm findings from the literature. Specifically, in the Netherlands, 4.3% of respondents reported great difficulty affording healthcare costs, while 10.4% and 12.5% reported difficulty and some difficulty affording healthcare costs, respectively. Furthermore, in the United Kingdom, the percentage of respondents reporting great difficulty, difficulty, and some difficulty affording healthcare costs was 1.9%, 3.2%, and 7.4%, respectively. In addition, in France, 2.3% of respondents reported great difficulty affording healthcare costs, while 7% and 12.5% reported difficulty and some difficulty affording healthcare costs, respectively. Finally, in Germany, the percentage of respondents reporting great difficulty, difficulty, and some difficulty affording healthcare costs was 2.4%, 4.3%, and 9.3%, respectively [117].

The economic crisis adversely affected a substantial percentage of the Greek population and negatively affected their capacity to satisfy healthcare needs. Indeed, the data from EU-SILC 2007 (Source: Hellenic Statistical Authority) indicate that the percentage of unmet health needs before starting the economic crisis, i.e., 2007, was 6.69%. In addition, among those reporting an unmet healthcare need, 68.98% declared affordability issues.

The economic crisis of 2008 affected the EU Member States in different ways. Taxation revenue decreased and borrowing costs increased in several member states. In some countries where healthcare is financed primarily through health insurance contributions from employees and employers, resources also decreased because of reduced wages and increasing unemployment rates. In addition, public expenditure increased because of an increase in take-up benefits such as unemployment benefits. In order to balance their budgets, many EU governments cut public expenditure including health expenditures. However, the crisis adversely impacted access to healthcare services not only because of budget cuts. Access to healthcare for households also reduced because disposable income decreased [118].

The mechanism on the basis of which economic crisis affected healthcare access is as follows: in combination with budget cuts, household income and wealth reduction led to a reduced ability to pay out of pocket. In countries with a work-related insurance system, job losses led to a loss of coverage. These in turn led to limited healthcare access [119].

Several high-and low-income countries in Europe, North America and Africa constitute examples of the abovementioned point [120,121,122,123,124].

Identifying the factors influencing healthcare affordability is particularly important because certain policies based on these factors can be applied during a period of recession especially for the more disadvantaged. However, this is not an easy task because affordability depends not only on the individuals’ characteristics but also on the characteristics of the healthcare system and their interrelation. In addition, as previously mentioned, healthcare affordability is a subjective concept.

The main limitations of this study are: (1) the lack of information with regard to insurance coverage; (2) the fact that the analysis of EQLS 2007 and EQLS 2016 was not based on weighted data, and (3) that the study almost exclusively focused on househlods’ and individuals’ characteristics. Except for the information on geographic characteristics, no information is available with regard to the health system (type of services, availability, etc.).

## 5. Conclusions

Access to healthcare in Greece presents challenges in terms of affordability, leading to high levels of unmet healthcare needs particularly among financially disadvantaged groups [125]. This point is of great importance because “the proof of access is use of service not simply the presence of a facility” [126].

However, because a large part of the world’s population has limited access to affordable healthcare [127], we argue that facing cost-related difficulties with regard to healthcare use is a global phenomenon. All people should have equal financial access to medical care [128], because the accessibility of appropriate and affordable healthcare is vital to improving health outcomes and reducing health disparities [129].

The provision of affordable healthcare services is definitely a challenge that is increasingly difficult. Due to the healthcare system complexities, investigating the utilization and costs of healthcare services is key to informed decision-making [130]. Policy makers must address a diverse array of issues to reduce health disparities by making healthcare more affordable and accessible [131] because achieving equity in health access and outcomes is one of the goals of health policy [132]. 

If we accept that access may be defined as freedom from barriers to healthcare [133], then understanding the full range of barriers for vulnerable populations such as the poor is important to optimizing healthcare and health outcomes [134].

Thus, there is a need for more research while considering the influence of healthcare systems and patient characteristics [59].

The results of this study confirm that healthcare affordability is a critical issue for the Greek population. Access improvement in Greece requires interventions with regard to healthcare system inefficiencies, but also improvements in living standards, meaning that several factors should be addressed. That is, a multi-factorial health, social, and economic policy approach is required.

The well-documented negative effect of the economic crisis on health outcomes can be partially attributed to the unaffordability of the healthcare services and the healthcare system characteristics.

## Figures and Tables

**Table 1:** Potential predictors (1st model).

Variable	Categories
Region	1: Attica
	2: Islands of Aegean and Crete
	3: Northern Greece
	4: Central Greece
Population Density	1: Thinly Populated Areas
	2: Intermediate Populated Areas
	3: Densely Populated Areas
Total Household Disposable Income	Continuous Variable
Ability to Make Ends Meet	1: With Great Difficulty
	2: With Difficulty
	3: With Some Difficulty
	4: Fairly Easily
	5: Easily
	6: Very Easily
Capacity to Cope with Unexpected Financial Expenses	0: No
	1: Yes
Being in Arrears on Utility Bills During the Last 12 Months	1: No
	2: Yes, Once
	3: Yes, Twice or More
Financial Burden Induced from Total Housing Cost	1: A Heavy Burden
	2: Somewhat of a Burden
	3: Not a Burden at All

**Table 2:** Potential predictors (2nd model).

Variable	Categories
Gender	0: Women
	1: Men
Age	1: 17–24
	2: 25–34
	3: 35–44
	4: 45–54
	5: 55–64
	6: 65–74
	7: 75+
Self-Rated Health	1: Very Bad
	2: Bad
	3: Moderate
	4: Good
	5: Very Good
Existence of a Chronic Health Condition	0: No
	1: Yes
Income	Continuous Variable
Education	1: Less than Primary Education
	2: Primary Education
	3: Lower Secondary Education
	4: Upper Secondary Education
	5: Post-Secondary, Non-Tertiary Education
	6: Short Cycle Tertiary Education
	7: Bachelor or Equivalent
	8: Master or Equivalent
	9: Doctorate or Equivalent
Occupation	1: Employee Working Full-time and Self-employed Working Full-time (Including Family Worker)
	2: Employee Working Part-time and Self-Employed Working Part-Time (Including Family Worker)
	3: Unemployed
	4: Pupil, Student, in Compulsory Military Community or Service, Further Training, Unpaid Work Experience
	5: In Retirement or in Early Retirement or Has Given Up Business
	6: Permanently Disabled or/and Unfit to Work
	7: Fulfilling Domestic Tasks and Care Responsibilities
	8: Other Inactive People

**Table 3:** Potential predictors (3rd model).

Variable	Categories
Degree of Urbanity (Subjective)	1: The Open Countryside
	2: A Village/Small Town
	3: A Medium to Large Town
	4: A City or City Suburb
Year	0: 2007
	1: 2016
Age	Continuous Variable
Gender	0: Women
	1: Men
Family Size	Continuous Variable
Self-Rated Health	1: Very Bad
	2: Bad
	3: Moderate
	4: Good
	5: Very Good
Existence of a Chronic Health Condition	0: No
	1: Yes
Education	1: Primary
	2: Secondary
	3: Tertiary
Occupation	1: At Work as Employee or Employer/Self-Employed
	2: Employed, on Childcare Leave
	3: Employed, on Other Special Leave (e.g., Sickness; Not Holiday)
	4: In Receipt of Retirement Pension and At Work as Employee or Employer/Self-Employed
	5: At work as Relative Assisting on Family Business or Farm
	6: Unemployed Less Than 12 Months
	7: Unemployed 12 Months or More
	8: Unable to Work Due to Long-Term Illness or disability
	9: Retired
	10: Full-Time Homemaker/Fulfilling Domestic Tasks
	11: In Education (at School, University, etc.)/Student
	12: Other
Ability to Make Ends Meet	1: Very Easily
	2: Easily
	3: Fairly Easily
	4: With Some Difficulty
	5: With Difficulty
	6: With Great Difficulty

**Table 4:** Healthcare affordability.

Responding to Healthcare Cost	Percent (%)
With Great Difficulty	31.97
With Difficulty	34.70
With Some Difficulty	22.72
Fairly Easily	7.67
Easily	2.58
Very Easily	0.36

**Table 5:** First model (logistic regression model).

Variable	OR	*p*	95% Confidence Interval	
Region		<0.001		
Islands of Aegean and Crete	0.45	<0.001	0.34	0.58
Northern Greece	0.58	<0.001	0.47	0.72
Central Greece	0.56	<0.001	0.44	0.70
Making Ends Meet		<0.001		
Making Ends Meet with Great Difficulty vs. Subsequent Levels	0.04	<0.001	0.03	0.05
Making Ends Meet with Difficulty vs. Subsequent Levels	0.04	<0.001	0.03	0.06
Making Ends Meet with Some Difficulty vs. Subsequent Levels	0.07	<0.001	0.04	0.11
Making Ends Meet Fairly Easily vs. Subsequent Levels	0.29	<0.001	0.15	0.57
Making Ends Meet Easily vs. Subsequent Level	0.57	0.377	0.16	2.00
Capacity to Cope with Unexpected Expenses	2.20	<0.001	1.80	2.69
Constant	0.40	<0.001	0.30	0.53

**Table 6:** Link test (first model).

Variable	Coefficient	*p*	95% Confidence Interval	
h	0.94	<0.001	0.83	1.05
$h^{2}$	−0.02	0.198	−0.06	0.01
Constant	0.01	0.899	−0.12	0.14

**Table 7:** Reasons for unmet medical need.

Reasons for Unmet Medical Need	Percent (%)
Could not Afford	83.15
Waiting List	6.13
Could not Take Time Because of Work, Care for Children or for Others etc.	2.56
Too Far to Travel or No Means of Transportation	1.37
Fear of Medical Doctors, Hospitals, Examination, or Treatment	1.22
Wanted to Wait and See if Problem got Better on its Own	4.84
Did not Know Any Good Medical Doctor	0.14
Other Reasons	0.59

**Table 8:** Second model (logistic regression model).

Variable	OR	*p*	95% Confidence Interval	
Education		<0.001		
Less than Primary Education vs. Subsequent Levels	2..19	<0.001	1.44	3.34
Primary Education vs. Subsequent Levels	2.33	<0.001	1.61	3.39
Lower Secondary Education vs. Subsequent Levels	3.15	<0.001	1.96	5.05
Upper Secondary Education vs. Subsequent Levels	2.38	<0.001	1.49	3.80
Post-Secondary Non-Tertiary Education vs. Subsequent Levels	1.58	0.173	0.82	3.03
Short Cycle Tertiary Education vs. Subsequent Levels	4.47	0.022	1.24	16.14
Bachelor or Equivalent vs. Subsequent Levels	3.48	0.007	1.40	8.67
Master or Equivalent vs. Subsequent Level	3.61	0.150	0.63	20.71
Occupation		<0.001		
Employee Working Part-Time and Self-Employed Working Part-Time (Including Family Worker)	1.70	0.058	0.98	2.93
Unemployed	3.69	<0.001	2.63	5.17
Pupil, Student, in Compulsory Military or Community Service, Further Training, Unpaid Work Experience	2.35	0.007	1.26	4.37
In Retirement or in Early Retirement or Has Given up Business	0.79	0.115	0.60	1.06
Permanently Disabled or/and Unfit to Work	1.78	0.084	0.93	3.43
Fulfilling Domestic Tasks and Care Responsibilities	1.67	0.001	1.23	2.27
Other Inactive People	2.10	0.169	0.73	6.02
Self-Rated Health		<0.001		
Very Bad vs. Subsequent Levels	0.57	0.004	0.39	0.84
Bad vs. Subsequent Levels	1.27	0.091	0.96	1.68
Fair vs. Subsequent Levels	1.45	0.003	1.14	1.85
Good vs. Subsequent Level	0.61	0.001	0.46	0.82
Constant	2.32	<0.001	1.71	3.14

**Table 9:** Link test (second model).

Variable	Coefficient	*p*	95% Confidence Interval	
h	1.21	<0.001	0.72	1.70
$h^{2}$	−0.07	0.364	−0.22	0.08
Constant	−0.13	0.500	−0.51	0.25

**Table 10:** Difficulty in responding to healthcare cost.

Difficulty in Responding to Healthcare Cost	2007	2016
Very Difficult	17.40	27.44
A little Difficult	28.82	37.41
Not Difficult at All	53.79	35.15

**Table 11:** Third model (logistic regression model).

Variable	OR	*p*	95% Confidence Interval	
Year	1.74	<0.001	1.44	2.10
Gender	0.83	0.049	0.69	1.00
Ability to Make Ends Meet		<0.001		
Very Easily vs. Subsequent Levels	0.30	0.003	0.13	0.66
Easily vs. Subsequent Levels	0.58	0.001	0.42	0.79
Fairly Easily vs. Subsequent Levels	0.43	<0.001	0.33	0.58
With Some Difficulty vs. Subsequent Levels	0.58	<0.001	0.47	0.72
With Difficulty vs. Subsequent Level	0.88	0.360	0.66	1.16
Constant	0.78	0.016	0.64	0.95

**Table 12:** Link test (third model).

Variable	Coefficient	*p*	95% Confidence Interval	
h	0.95	<0.001	0.77	1.13
$h^{2}$	0.18	0.188	−0.09	0.44
Constant	−0.05	0.419	−0.18	0.07
